# MRI-Based Radiomics Models for Predicting Risk Classification of Gastrointestinal Stromal Tumors

**DOI:** 10.3389/fonc.2021.631927

**Published:** 2021-05-10

**Authors:** Haijia Mao, Bingqian Zhang, Mingyue Zou, Yanan Huang, Liming Yang, Cheng Wang, PeiPei Pang, Zhenhua Zhao

**Affiliations:** ^1^ Department of Radiology, Shaoxing People’s Hospital, Shaoxing Hospital, Zhejiang University School of Medicine, Shaoxing, China; ^2^ Department of Pathology, Shaoxing People’s Hospital, Shaoxing Hospital, Zhejiang University School of Medicine, Shaoxing, China; ^3^ Department of Pharmaceuticals Diagnosis, GE Healthcare, Hangzhou, China

**Keywords:** gastrointestinal stromal tumour, radiomics, classification, magnetic resonance imaging, model

## Abstract

**Background:**

We conduct a study in developing and validating four MRI-based radiomics models to preoperatively predict the risk classification of gastrointestinal stromal tumors (GISTs).

**Methods:**

Forty-one patients (low-risk = 17, intermediate-risk = 13, high-risk = 11) underwent MRI before surgery between September 2013 and March 2019 in this retrospective study. The Kruskal–Wallis test with Bonferonni correction and variance threshold was used to select appropriate features, and the Random Forest model (three classification model) was used to select features among the high-risk, intermediate-risk, and low-risk of GISTs. The predictive performance of the models built by the Random Forest was estimated by a 5-fold cross validation (5FCV). Their performance was estimated using the receiver operating characteristic (ROC) curve, summarized as the area under the ROC curve (AUC). Area under the curve (AUC), accuracy, sensitivity, and specificity for risk classification were reported. Linear discriminant analysis (LDA) was used to assess the discriminative ability of these radiomics models.

**Results:**

The high-risk, intermediate-risk, and low-risk of GISTs were well classified by radiomics models, the micro-average of ROC curves was 0.85, 0.81, 0.87 and 0.94 for T1WI, T2WI, ADC and combined three MR sequences. And ROC curves achieved excellent AUCs for T1WI (0.85, 0.75 and 0.82), T2WI (0.69, 0.78 and 0.78), ADC (0.85, 0.77 and 0.80) and combined three MR sequences (0.96, 0.92, 0.81) for the diagnosis of high-risk, intermediate-risk, and low-risk of GISTs, respectively. In addition, LDA demonstrated the different risk of GISTs were correctly classified by radiomics analysis (61.0% for T1WI, 70.7% for T2WI, 83.3% for ADC, and 78.9% for the combined three MR sequences).

**Conclusions:**

Radiomics models based on a single sequence and combined three MR sequences can be a noninvasive method to evaluate the risk classification of GISTs, which may help the treatment of GISTs patients in the future.

## Introduction

Gastrointestinal stromal tumors (GISTs) is a rare sarcoma of soft tissue that can occur anywhere in the gastrointestinal tract, affecting 6–20 people per million per year in Western and Asian countries (1, 2). GISTs originates from the interstitial cells (ICC) of Cajal or common precursor cells (3). Surgical resection is the gold standard for the treatment of gastrointestinal stromal tumors, but as the risk of tumors increases, the risk of postoperative recurrence also increases (4, 5). At present, the recognized standard for risk classification of GISTs is the National Institutes of Health (revised in 2008), which can be classified as high-risk, intermediate-risk, low-risk and very low-risk, according to tumor size, mitotic index, and primary tumor site (6). Studies have shown that NIH classification has important prognostic value (5). The survival rate of high-risk GISTs patients is significantly worse than that of intermediate-risk or low/very low-risk GISTs patients (7). However, pathological evaluation of these surgical specimens is applied postoperatively because it is difficult to calculate the mitotic count before surgery. Therefore, it is still difficult to classify the risk of GISTs before operation. However, for high-risk GISTs patients, previous studies have shown that preoperative targeted drug therapy, such as Imatinib, can shrink the tumor and limit the scope of surgical resection, and improve the prognosis of patients with GISTs (8, 9). Therefore, accurate preoperative assessment the risk of GISTs has high clinical value, which can provide important clues for predicting the prognosis of the disease and the use of adjuvant chemotherapy.

In recent years, with the development and application of radiomics, hundreds of standardized and quantifiable imaging features can be extracted from CT/MRI images to assess the biological behavior of a tumor comprehensively, which may potentially improve the accuracy of diagnosis, prognosis and prediction (10). Previous studies used the subjective manifestations of lesions on CT images (tumor size, shape, CT density, enhancement mode, etc.), CT functional parameters, fractal analysis and CT-based radiomics to assess the risk classification of GISTs (11–14). Large differences between observers shown in subjective signs of imaging in the judgment of GISTs risk classification, due to the different experience of imaging doctors and poor repeatability of subjective signs. Besides, CT fractal analysis can be influenced by various factors such as noise, window width and level, and setting of the software (15). CT-based radiomics have obtained good results in the risk classification of GISTs. However, compared with MR multi-sequence imaging, it provides relatively limited texture features. Studies have used SUVmax in PET/CT to assess the risk of GISTs, it’s clinical application value is relatively low due to the high price and long examination time (16). MR imaging, as a non-ionizing radiation examination compared with CT, can provide more lesion information through multi-sequence imaging in evaluating the biological behavior of abdominal tumors (17). DWI can reflect the dispersion and movement restriction of water molecules. Some studies have shown that DWI texture features can be used as a biological indicator to evaluate the heterogeneity and prognosis of metastatic GISTs (18). Therefore, this study will use DWI texture analysis to study the heterogeneity of GISTs in water molecular dispersion. As a comparison, we will also study the effectiveness of the risk classification of GISTs in the T1, T2 sequence and combined three MR sequences. The purpose of this study was to establish MRI-based radiomics models for noninvasive assessment of GISTs risk classification.

## Materials and Methods

### Study Participants

The institutional review board of our hospital approved this retrospective study and waived the requirement to obtain patient approval or written informed consent for the review of medical records or images.

We enrolled 47 patients with Gastrointestinal stromal tumors (GISTs) from our center from September 2013 and March 2019. The inclusion criteria were as follows: (1) patients who underwent surgery for GISTs with curative intent; (2) patients underwent MR less than 15 days before surgical resection; (3) patients with complete clinicopathologic data. The exclusion criteria were as follows: (1) patients received imatinib therapy or other tyrosine kinase inhibitor as a neoadjuvant before surgery (n=4); (2) ADC sequence image was missing (n=2). Finally, 41 patients were included in our study.

Demographic and clinicopathologic data, including age, gender, primary tumor site, size of the tumor (maximum diameter) and mitotic count, were derived from medical records. The NIH modified criteria (**Table 1**) were used to stratify the malignant potential of GISTs on the basis of the clinical and postoperative histological index, as a verification of our model. Studies have shown that according to the NIH standards, there is no significant prognostic difference between the very low-risk group and low-risk group (7). Therefore, we combined the very low-risk and low-risk into one group (low-risk group). Finally, our study includes three groups (low-risk, intermediate-risk, and high-risk).

**Table 1:** NIH 2008 criteria for defining risk stratification of GISTs recurrence after surgery.

Risk category	Tumor size (cm)	Mitotic index (per 50 HPF)	Primary tumor site
Very low risk	≤ 2.0	≤ 5.0	Any
Low risk	2.1–5.0	≤ 5.0	Any
Intermediate risk	≤ 5.0	6–10	Gastric
	5.1–10.0	≤ 5.0	Gastric
High risk	> 10.0	Any	Any
	Any	> 10	Any
	> 5.0	> 5	Any
	≤ 5.0	> 5	Non-gastric
	5.1–10.0	≤ 5	Non-gastric

NIH, National Institutes of Health.

### MRI Protocol

The MR examination was performed using a Verio 3.0T system (Siemens, Germany) with a dedicated twelve-channel abdomen coil. The routine protocol was composed of spin-echo T1-weighted (repetition time msec/echo time msec, 220/2; matrix, 208 × 256; field of view, 38 cm; slice thickness, 4 mm), T2-weighted fat-suppressed spin-echo (6647/81; matrix, 256 × 320; field of view, 38 cm; slice thickness, 6 mm) sequences, and axial DWI (7400/73; matrix, 220 × 292; field of view, 38 cm; slice thickness, 6 mm) with b values of 0 and 600 sec/mm^2^.

### MR Radiomics Analysis

**Figure 1:**
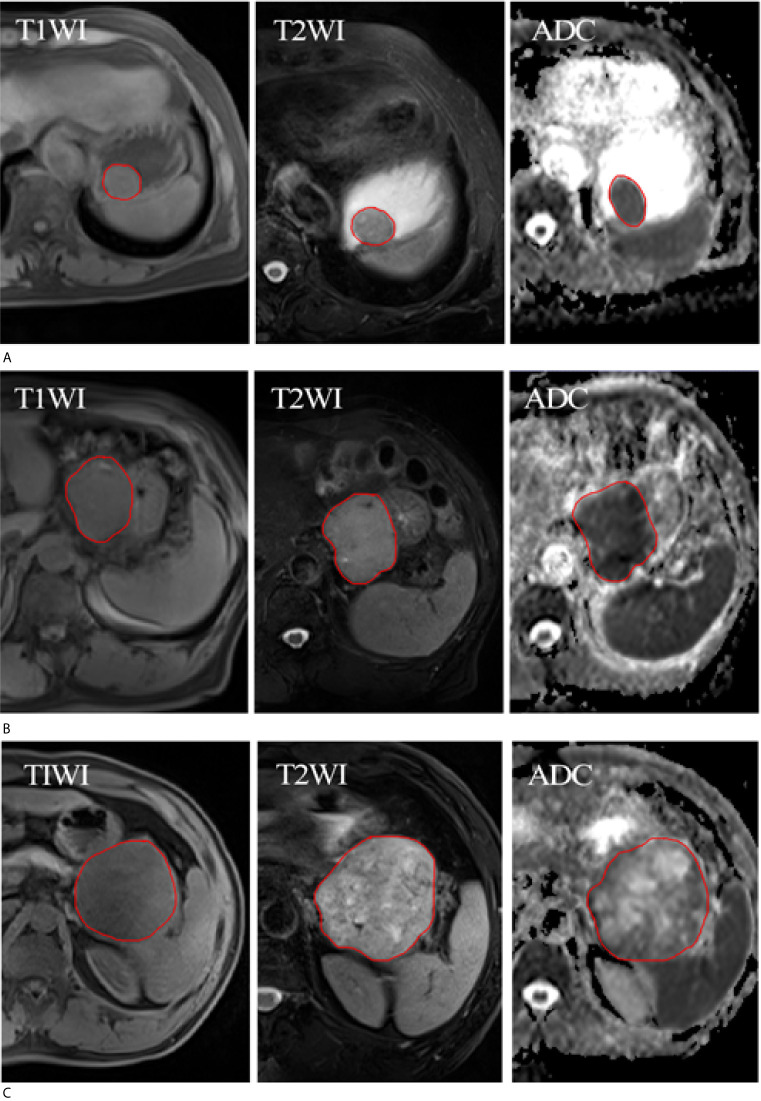
ROI of three patients with different risk classification of GISTs. **(A)** Three MR sequences (T1WI, T2WI, and ADC) show a low-risk of GISTs in a 68-year-old woman. **(B)** Three MR sequences show a intermediate-risk of GISTs in a 78-year-old man. **(C)** Three MR sequences show a high-risk of GISTs in a 50-year-old man. The red circle represents the ROI.

**Figure 2:**
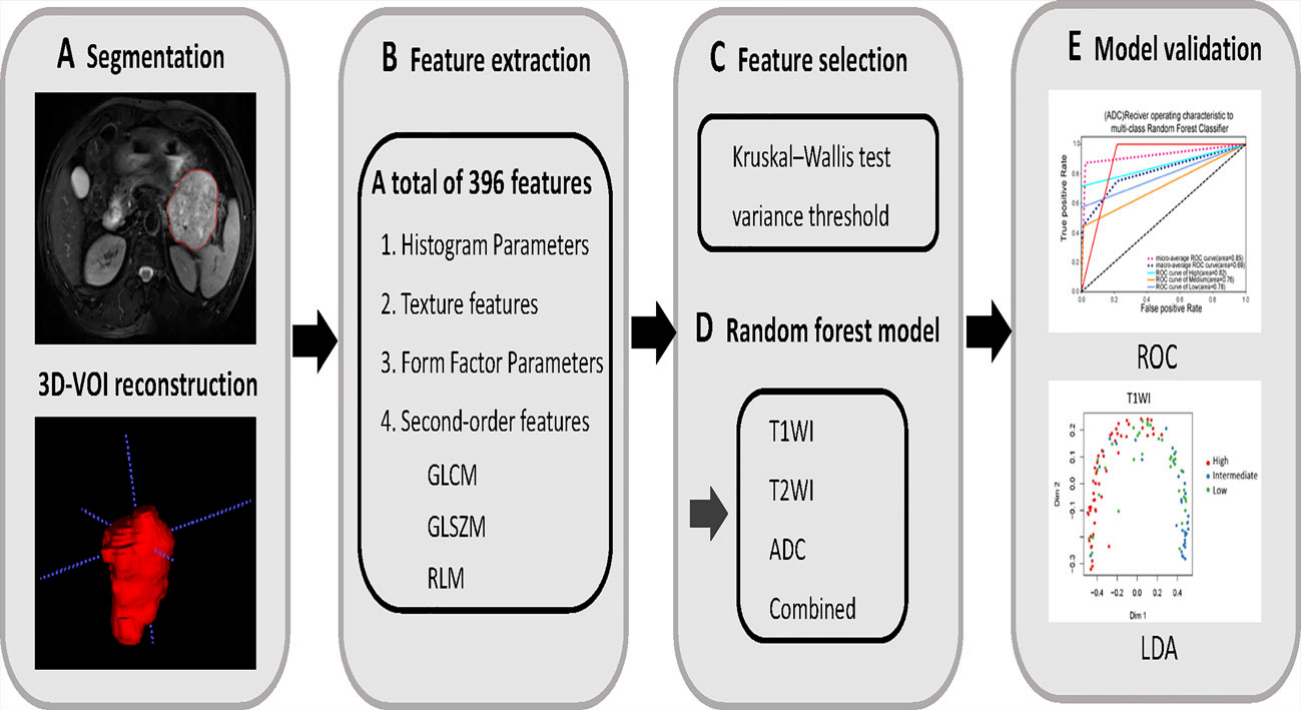
**(A)** MR images segmentation. The ITK-SNAP software was used to manually outline and segment the tumor region by slices, and then 3D-VOI reconstruction was performed to extract the texture parameters. **(B)** Texture features extraction. According to the segmentation image, a total of 396 texture parameters of different risk of GISTs were extracted from each set of images. **(C)** Texture features selection. After the parameters were normalized and dimensionality reduced, the characteristic parameters were selected by Kruskal–Wallis test and variance threshold. **(D, E)** Model establishment. Using random forest to build multi-sequence MRI model and its diagnostic efficacy was evaluated by ROC and LDA analysis.

After patients’ MR images were collected, the region of interest (ROI) were contoured manually on three MR sequences (T1WI, T2WI, and ADC), respectively. Two experienced abdominal radiologists, including a 10 years experienced radiologist (depict the ROI) and a 15 years experienced radiologist (check the ROI), outlined each layer of the lesion to form a 3D ROI and saved in a 3D format by using ITK-snap (Version3.8.0, www.itksnap.org, **Figure 1**) (19). Then, the imaging features were extracted using AK (Artificial Intelligent Kit, GE Healthcare, China). Finally, a total of 396 features were extracted from the analysis of the volumes inspected. These parameters included Histogram Parameters (Energy, Entropy, MaxIntensity, MinIntensity, MeanValue, FrequencySize, VolumeCount, etc); Texture features (Skewness, Kurtosis, Correlation, Cluster Shade, Cluster Prominence); Form Factor Parameters (Sphericity, Surface area, Compactness, Maximum 3D diameter, Spherical disproportion) and second-order features (gray level co-occurrence matrix, GLCM; gray level size zone matrix, GLSZM; gray level run-length matrix, GLRLM). All these radiomics features were further analyzed within the entire cohort of 41 patients. **Figure 2** describes the texture parameter extraction process.

### Statistical Analysis

Continuous variables are summarized with medians and ranges; categorical variables are described with frequencies and percentages. The patients’ clinical characteristics among the three groups were analyzed with the chi-square test.

Using the Kruskal–Wallis test, differences among the three groups’ radiomics features were measured in the T1WI, T2WI, and ADC sequences; p values were adjusted using Bonferonni correction and p values less than 0.017 (0.05/3) were considered statistically significant. And then, the variance threshold algorithm was used to remove those radiomics features with low variances. Hence, the appropriate feature sizes were selected by feature selection methods (Kruskal–Wallis test and variance threshold). Using these selected radiological characteristics, the Random Forest model is used for analysis (three classification model) to determine whether the selected characteristics can distinguish different risk classification. The predictive performance of the models built by the Random Forest was estimated by a 5-fold cross validation (5FCV). Their performance was estimated using the receiver operating characteristic (ROC) curve, summarized as the area under the ROC curve (AUC). The cohort was randomly split into five subsamples: one formed the test dataset for verifying the effectiveness of the model, and the others formed the training dataset to determine risk classification of GISTs for the model. The cross-validation process was repeated five times, with each of the five subsamples used as the validation data once. For selecting the best features, their performance was estimated using the receiver operating characteristic (ROC) curve, summarized as the area under the ROC curve (AUC). Ranked by AUC, the 30 most important features of all features were used to train the classifier. In addition, the AUC, accuracy, specificity and sensitivity at the best cut-off point, and 95% confidence interval are also demonstrated. Since the results of our study are multi-category indicators, we use micro-averaging of ROC to make statistics on each example in the data set regardless of category, to evaluate the effectiveness of the model. At the same time, LDA (multiclassification model) and LOOCV (Leave-one-out cross- validation) was performed to evaluate and verify the discrimination ability of the single and combined sequences models on the basis of the selected radiomics features by above method.

Statistical analyses for the present study were performed with R (version 3.5.1). A two-sided p value < 0.05 indicated statistical significance.

## Results

### Patient Characteristics

Forty-one patients were comprised of men (19 cases) and women (22 cases), gastric (32 cases) and non-gastric (9 cases), the low-risk (17 cases, 66.4 years, range 49-84 years), the intermediate-risk (13 cases, 71.2 years, range 59-85years), and the high-risk (11 cases, 65.0 years, range 47-87 years). A statistical difference in tumor size and mitotic number among the three groups (P <0.001, P =0.002) was found, but no statistical differences were found in age (P = 0.249), gender (P = 0.360), primary tumor site (P = 0.252) among the three groups. The clinicopathologic characteristics of gender, age, primary tumor site, risk classification, and mitotic count of the three groups were summarized in **Table 2**.

**Table 2:** Baseline characteristics of patients.

	Low-risk (17) Median (Range or %)	Intermediate-risk (13) Median (Range or %)	High-risk (11) Median (Range or %)	P Value
Age	66.4(49-84)	71.2(59-85)	65.0(47-87)	0.249
Gender Female Male	11(64.7)6(35.3)	7(53.8)6(46.2)	4(36.4)7(63.6)	0.360
Tumor size(mm)	35.5(22-50)	66.3(33-100)	98.7(25-206)	<0.001*
Location Gastric Non-Gastric	13(76.5)4(23.5)	12(92.3)1(7.7)	7(63.6)4(36.4)	0.252
Mitotic index (per 50 HPF) ≤5 >5	17(100)0	12(92.3)1(7.7)	6(54.5)5(45.5)	0.002*

P value is derived from the univariable association analyses between each characteristic and potential malignancy. Analysis of Variance was applied in continuous variables. The chi-square test was applied in categorical variables GISTs. HPF, high-power field. *P value < 0.05.

### Selection of Extracted Radiomics Features and Performance of Risk Classification

**Figure 3:**
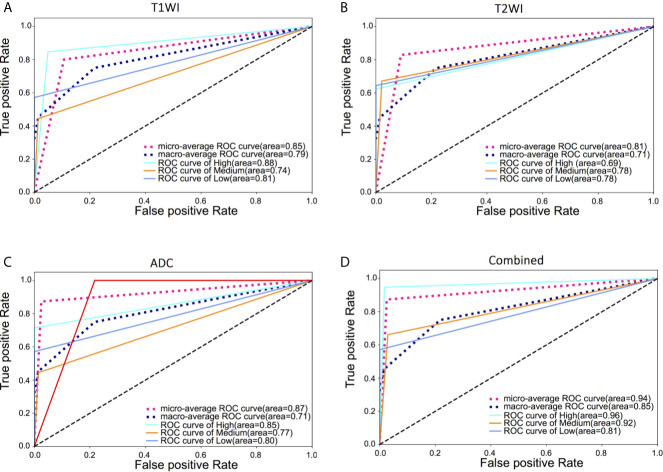
ROC curves based on radiomics features extracted from three MRI sequences and combined three MRI sequences for classification of three risks of GISTs. **(A)** micro-average of 0.85 on T1WI. AUC of 0.88, 0.74, and 0.81 for the diagnosis of high-risk, intermediate-risk, and low-risk, respectively; **(B)** micro-average of 0.81 on T2WI. AUC of 0.69, 0.78, and 0.78 for the diagnosis of high-risk, intermediate-risk, and low-risk, respectively; **(C)** micro-average of 0.87 on ADC. AUC of 0.85, 0.77, and 0.80 for the diagnosis of high-risk, intermediate-risk, and low-risk, respectively; **(D)** micro-average of 0.94 in combined three MR sequences and AUC of 0.96, 0.92, and 0.81 for the diagnosis of high-risk, intermediate-risk, and low-risk, respectively.

In the MR images of three sequences (T1WI, T2WI, and ADC), the thirty most important parameters based on contribution to classification in Random Forest for each sequence are shown in **Supplement**. Radiomics features such as Grey Level Nonuniformity, Run Length Nonuniformity, Volume were significantly different among the three GISTs risks groups on three sequences. By using ROC curves after 5FCV selecting features, the effectiveness of these selected features for the risk classification of GISTs was tested. As a result, we obtained a micro-average of 0.85 on T1WI. Besides, AUC of 0.85 (95% CI: 0.91, 0.98), 0.75 (95% CI: 0.78, 0.89), and 0.82 (95% CI: 0.87, 0.90) for the diagnosis of high-risk, intermediate-risk, and low-risk, respectively. With T2WI images, we obtained a micro-average of 0.81. As for AUC of 0.69 (95% CI: 0.88, 0.94), 0.78 (95% CI: 0.74, 0.81), and 0.78 (95% CI: 0.83, 0.96) for the diagnosis of high-risk, intermediate-risk, and low-risk, respectively. For ADC MR images, we obtained a micro-average of 0.87 and AUC of 0.85 (95% CI: 0.79, 0.90),0.77 (95% CI: 0.86, 0.94), and 0.80 (95% CI: 0.85, 0.92) for the diagnosis of high-risk, intermediate-risk, and low-risk, respectively. In combined three MR sequences, micro-average of showed a slight increase to 0.94 compared with any MR sequence alone for differentiating risks of GISTs. Shown in **Tables 3** **,** **4** and **Figure 3**.

**Table 3:** ROC of three single sequences for distinguishing different risk of GISTs.

	Status	AUC	ACC	Sensitivity	Specificity	95% confidence interval of AUC	
						Lower bound	Upper bound
T1WI	High	0.85	0.91	0.89	0.91	0.91	0.98
	Intermediate	0.75	0.71	0.82	0.79	0.78	0.89
	Low	0.82	0.86	0.96	0.85	0.87	0.90
T2WI	High	0.69	0.92	0.77	0.99	0.88	0.94
	Intermediate	0.78	0.95	0.81	0.98	0.74	0.81
	Low	0.78	0.82	0.93	0.95	0.83	0.96
ADC	High	0.85	0.82	0.73	0.81	0.79	0.90
	Intermediate	0.77	0.90	0.92	0.85	0.86	0.94
	Low	0.80	0.76	0.89	0.82	0.85	0.92

**Table 4:** Classification performance of four models for GIST risk level.

Radiomics Models	Micro-average
T1 sequence	0.85
T2 sequence	0.81
ADC sequence	0.87
Combined three MR sequences	0.94

**Figure 4:**
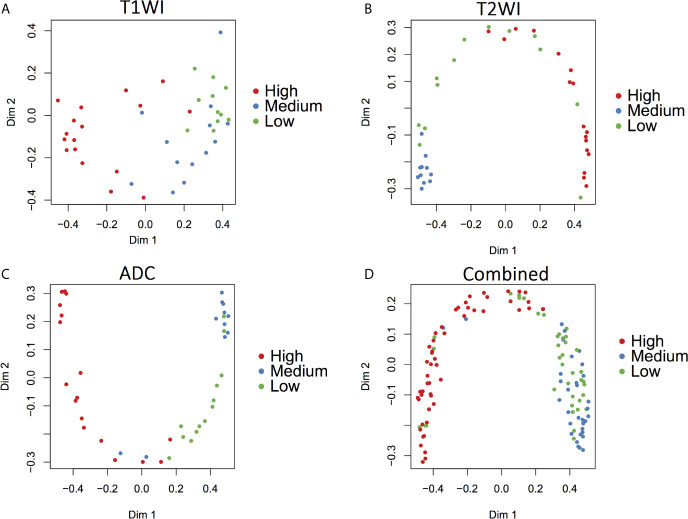
Discriminant function analysis based on radiomics features extracted from three MRI sequences for the GISTs risk classification. **(A)** T1WI, 61.0% of the three originally grouped cases (three GISTs risk classification) were correctly classified; **(B)** T2WI, 70.7% of the three originally grouped cases (three GISTs risk classification) were correctly classified; **(C)** ADC, 83.3% of the three originally grouped cases (three GISTs risk classification) were correctly classified; **(D)** combined three MR sequences, 78.9% of the three originally grouped cases (three GISTs risk classification) were correctly classified.

LDA was then used to assess the discriminative ability of these selected radiomics features, while LOOCV was used to correct the result. (**Figure 4**). For T1WI sequence, 61.0% of the three originally grouped cases (three GISTs risk groups) were correctly classified, while 58.5% of these three grouped cases abovementioned were correctly classified after cross-validation. For T2WI sequence, 70.7% of the originally grouped cases were correctly classified and 58.5% of the cross-validated grouped cases were correctly classified. As for ADC sequence, 83.3% of the originally grouped cases were correctly classified and 66.2% of the cross-validated grouped cases were correctly classified. When combining three MR sequences,78.9% of the three originally grouped cases (three GISTs risk groups) were correctly classified, while 65.0% of these three grouped cases abovementioned were correctly classified after cross-validated.

## Discussion

In this study, we evaluated the diagnostic value of radiomic features extracted from MR images in identifying the risk of GISTs (low-risk, intermediate-risk, and high-risk). Previous studies have mostly used imaging findings, such as necrotic cysts, to assess the risk of GISTs, but the precise staging of stromal tumor aggressiveness has great differences between observers and limited accuracy (20–22).We found that some radiomics features were significantly different among the three risk classifications of GISTs. Based on these radiomics features, the ROC curve yielded decent AUC. This result indicated that our MR-based radiomics method yielded excellent performance in distinguishing low-risk, intermediate-risk and high-risk GISTs. Considering that all three sequences in our study are routinely used in our center, our results have good clinical application prospects.

We found several interesting points of significantly different radiomics features among the three levels of risk of GISTs. For example, among T1WI, T2WI and ADC sequences, Grey Level Nonuniformity, Volume, Run Length Nonuniformity, and Frequency Size have significant specificity among the three levels of risk of GISTs. These findings abovementioned might indicate greater textural heterogeneity on high risk GISTs, which were consistent with previous studies (23, 24). Ren et al. aimed to predict the malignant potential assessment of GISTs patients through CT texture features before surgery and found that high malignant potential GISTs demonstrated obviously higher heterogeneity than low malignant potential GISTs demonstrated (23). Furthermore, Yang et al. constructed a nomogram based on MR radiomics features such as RunLengthNonUniformity, ShortRunHighGrayLevelEmphasis, and OriginalFirstorderMinimum. The calculated scores demonstrated that high malignant potential GISTs was significantly more heterogeneous than low malignant potential (24). In addition, among the features we selected, the parameters reflecting the shape of the lesion, such as volume and maximum 3D diameter on MRI has value for guiding GISTs risk classification, which is consistent with tumor size as an important factor in assessing the malignant potential and prognosis of GISTs (see **Table 1**).

We found that the ADC sequence outperformed T1WI and T2WI sequences in evaluating the risk classification of GISTs. In ADC sequence, the AUC of high-risk group was 0.85, which indicated that the ADC sequence had high efficiency for high-risk group identification. To date, some studies have evaluated the discriminative ability of different MRI sequences on the basis of radiomics, among which some studies have mentioned to the favorable predictive value of ADC in radiomics analyses on discriminating benign and malignant tumors (25, 26). The radiomics model based on ADC sequence has a positive application in the classification of meningioma, cholangiocarcinoma and glioma (27–30).

In our study, we have proven that our combined three MR sequences radiomics model has excellent performance in diagnosing different risk classifications of GISTs correctly (micro-average=0.94), especially in identifying high-risk GISTs (AUC=0.96). Wang et al. (31)used CT images to establish a predictive model to distinguish the high and low malignant potential of GISTs and the AUC of the model was 0.882. The results show that the feature extraction of multi sequence MR images can provide more texture information of lesions, which is helpful to improve the ability of the model to evaluate the risk of GISTs (32–34). In addition, our study also employed an uncommon statistical method (LDA) to assess the diagnostic ability of radiomics models as a supplement to ROC curve, which provides a new perspective for evaluating radiomics data.

However, our study had several limitations although the results were encouraging. First, compared with a large number of extracted radiological features, the sample size of our study is relatively small. Therefore, we use Random Forest to avoid overfitting in the model derivation process (35), and in the future can improve by increasing the sample size. Therefore, large-scale, prospective and multi-center studies are needed to validate our results. Second, due to the large slice thickness and interslice gap in MR imaging, it is easy to cause the partial volume effect of small tumors. Therefore, tumors size less than 2 cm were excluded from our study. In future, we consider reducing the thickness of the slice to facilitate the inclusion of small tumors with a maximum diameter of 1.0-2.0 cm. Third, we did not consider gene mutations in this study, such as KIT and PDGFRA mutations (36, 37), which are essential for diagnosing some difficult cases, predicting the therapeutic effect of targeted drugs and guiding medical decision-making. Therefore, we will consider genome characteristics to build a more comprehensive radiogenomics model in the future.

## Conclusion

In conclusion, our research proposes that radiomics models based on a single sequence and combination of multiple sequences can help classify the risk of GISTs. As a noninvasive and reproducible method, radiomic analysis may become a potential biomarker for GISTs. If finally put into practice, it may completely change the diagnosis and clinical treatment of GISTs, although it still has a long way to go.

## Data Availability Statement

The original contributions presented in the study are included in the article/**Supplementary Material**. Further inquiries can be directed to the corresponding author.

## Ethics Statement

The studies involving human participants were reviewed and approved by Ethics Committee of Shaoxing People’s Hospital. Written informed consent for participation was not required for this study in accordance with the national legislation and the institutional requirements. Written informed consent was not obtained from the individual(s) for the publication of any potentially identifiable images or data included in this article.

## Author Contributions

HM and BZ conceived and designed this study. MZ conducted the study and collected important background data. YH, MZ, and BZ helped to collect the clinical data. HM and BZ drafted the manuscript. LY performed some image scanning and processing. CW performed the histological examination. ZZ and PP put forward many opinions on the manuscript. All authors contributed to the article and approved the submitted version.

## Funding

This study was supported in part by grants from Medical and Health Research Project of Zhejiang Province (grant number, 2021KY370), Medical and Health Research Project of Zhejiang Province (grant number, 2021KY1135), Public Welfare Technology Application Research Project of Zhejiang Province (grant number, LGF19H220002) and institution from Key Laboratory of Functional Molecular Imaging of Tumor and Interventional Diagnosis and Treatment of Shaoxing City (Shaoxing People’s Hospital, Shaoxing, Zhejiang, China).

## Conflict of Interest

Author PP was employed by company GE Healthcare.

The remaining authors declare that the research was conducted in the absence of any commercial or financial relationships that could be construed as a potential conflict of interest.

## Abbreviations

GISTs, Gastrointestinal stromal tumors; ROC, Receiver operating characteristic; AUC, Area under the curve; MRI, Magnetic resonance images; GLCM, Gray level co-occurrence matrix; GLSZM, Gray level size zone matrix; GLRLM, Gray level run-length matrix; LDA, Linear discriminant analysis; ROI, Region of interest.

## References

[1] Akahoshi K Oya M Koga T Shiratsuchi Y. Current Clinical Management of Gastrointestinal Stromal Tumor. World J Gastroenterol (2018) 24:2806–17. 10.3748/wjg.v24.i26.2806 PMC6048423 30018476

[2] van der Graaf WTA Tielen R Bonenkamp JJ Lemmens V Verhoeven RHA de Wilt JHW. Nationwide Trends in the Incidence and Outcome of Patients With Gastrointestinal Stromal Tumour in the Imatinib Era. Br J Surg (2018) 105:1020–7. 10.1002/bjs.10809 PMC6033139 29664995

[3] Parab TM DeRogatis MJ Boaz AM Grasso SA Issack PS Duarte DA. Gastrointestinal Stromal Tumors: A Comprehensive Review. J Gastrointest Oncol (2019) 10:144–54. 10.21037/jgo.2018.08.20 PMC6351301 30788170

[4] Plaat BE Hollema H Molenaar WM Torn Broers GH Pijpe J Mastik MF. Soft Tissue Leiomyosarcomas and Malignant Gastrointestinal Stromal Tumors: Differences in Clinical Outcome and Expression of Multidrug Resistance Proteins. J Clin Oncol (2000) 18:3211–20. 10.1200/JCO.2000.18.18.3211 10986053

[5] Joensuu H Vehtari A Riihimaki J Nishida T Steigen SE Brabec P. Risk of Recurrence of Gastrointestinal Stromal Tumour After Surgery: An Analysis of Pooled Population-Based Cohorts. Lancet Oncol (2012) 13:265–74. 10.1016/S1470-2045(11)70299-6 22153892

[6] Joensuu H. Risk Stratification of Patients Diagnosed With Gastrointestinal Stromal Tumor. Hum Pathol (2008) 39:1411–9. 10.1016/j.humpath.2008.06.025 18774375

[7] Zhao B Zhang J Mei D Zhang J Luo R Xu H. The Assessment of Different Risk Classification Systems for Gastrointestinal Stromal Tumors (GISTs): The Analytic Results From the SEER Database. Scand J Gastroenterol (2018) 53:1319–27. 10.1080/00365521.2018.1515319 30353759

[8] Mantese G. Gastrointestinal Stromal Tumor: Epidemiology, Diagnosis, and Treatment. Curr Opin Gastroenterol (2019) 35:555–9. 10.1097/MOG.0000000000000584 31577561

[9] Joensuu H Eriksson M Sundby Hall K Reichardt A Hartmann JT Pink D. Adjuvant Imatinib for High-Risk GI Stromal Tumor: Analysis of a Randomized Trial. J Clin Oncol (2016) 34:244–50. 10.1200/JCO.2015.62.9170 26527782

[10] Verma V Simone CB,2 Krishnan S Lin SH Yang J Hahn SM. The Rise of Radiomics and Implications for Oncologic Management. J Natl Cancer Inst (2017) 109(7). 10.1093/jnci/djx055 28423406

[11] Choi IY Yeom SK Cha J Cha SH Lee SH Chung HH. Feasibility of Using Computed Tomography Texture Analysis Parameters as Imaging Biomarkers for Predicting Risk Grade of Gastrointestinal Stromal Tumors: Comparison With Visual Inspection. Abdom Radiol (New York) (2019) 44:2346–56. 10.1007/s00261-019-01995-4 30923842

[12] Su Q Wang Q Zhang H Yu D Wang Y Liu Z. Computed Tomography Findings of Small Bowel Gastrointestinal Stromal Tumors With Different Histologic Risks of Progression. Abdom Radiol (New York) (2018) 43:2651–8. 10.1007/s00261-018-1511-6 29492604

[13] Zhang X Bai L Wang D Huang X Wei J Zhang W. Gastrointestinal Stromal Tumor Risk Classification: Spectral CT Quantitative Parameters. Abdom Radiol (New York) (2019) 44:2329–36. 10.1007/s00261-019-01973-w 30980116

[14] Jiang ZX Zhang SJ Peng WJ Yu BH. Rectal Gastrointestinal Stromal Tumors: Imaging Features With Clinical and Pathological Correlation. World J Gastroenterol (2013) 19:3108–16. 10.3748/wjg.v19.i20.3108 PMC3662951 23716991

[15] Kurata Y Hayano K Ohira G Narushima K Aoyagi T Matsubara H. Fractal Analysis of Contrast-Enhanced CT Images for Preoperative Prediction of Malignant Potential of Gastrointestinal Stromal Tumor. Abdom Radiol (New York) (2018) 43:2659–64. 10.1007/s00261-018-1526-z 29500645

[16] Yoshikawa K Shimada M Kurita N Sato H Iwata T Morimoto S. Efficacy of PET-CT for Predicting the Malignant Potential of Gastrointestinal Stromal Tumors. Surg Today (2013) 43:1162–7. 10.1007/s00595-012-0411-6 23143169

[17] Low RN Semelka RC Worawattanakul S Alzate GD Sigeti JS. Extrahepatic Abdominal Imaging in Patients With Malignancy: Comparison of MR Imaging and Helical CT, With Subsequent Surgical Correlation. Radiology (1999) 210:625–32. 10.1148/radiology.210.3.r99mr46625 10207459

[18] Fu J Fang M-J Dong D Li J Sun Y-S Tian J. Heterogeneity of Metastatic Gastrointestinal Stromal Tumor on Texture Analysis: DWI Texture as Potential Biomarker of Overall Survival. Eur J Radiol (2020) 125:108825. 10.1016/j.ejrad.2020.108825 32035324

[19] Yushkevich PA Piven J Hazlett HC Smith RG Ho S Gee JC. User-Guided 3D Active Contour Segmentation of Anatomical Structures: Significantly Improved Efficiency and Reliability. Neuroimage (2006) 31:1116–28. 10.1016/j.neuroimage.2006.01.015 16545965

[20] Wang JK. Predictive Value and Modeling Analysis of MSCT Signs in Gastrointestinal Stromal Tumors (GISTs) to Pathological Risk Degree. Eur Rev Med Pharmacol Sci (2017) 21:999–1005. 28338197

[21] Vernuccio F Taibbi A Picone D LAG L Midiri M Lagalla R. Imaging of Gastrointestinal Stromal Tumors: From Diagnosis to Evaluation of Therapeutic Response. Anticancer Res (2016) 36:2639–48. 27272772

[22] Zhou C Duan X Zhang X Hu H Wang D Shen J. Predictive Features of CT for Risk Stratifications in Patients With Primary Gastrointestinal Stromal Tumour. Eur Radiol (2016) 26:3086–93. 10.1007/s00330-015-4172-7 26699371

[23] Ren C Wang S Zhang S. Development and Validation of a Nomogram Based on CT Images and 3D Texture Analysis for Preoperative Prediction of the Malignant Potential in Gastrointestinal Stromal Tumors. Cancer Imaging (2020) 20:5. 10.1186/s40644-019-0284-7 31931874 PMC6958787

[24] Yang L Zheng T Dong Y Wang Z Liu D Du J. MRI Texture-Based Models for Predicting Mitotic Index and Risk Classification of Gastrointestinal Stromal Tumors. J Magn Reson Imaging (2020) 53:e27390. 10.1002/jmri.27390 33037745

[25] Surov A Meyer HJ Wienke A. Can Apparent Diffusion Coefficient (ADC) Distinguish Breast Cancer From Benign Breast Findings? A Meta-Analysis Based on 13 847 Lesions. BMC Cancer (2019) 19:955. 10.1186/s12885-019-6201-4 31615463 PMC6794799

[26] Xu M Fang M Zou J Yang S Yu D Zhong L. Using Biparametric MRI Radiomics Signature to Differentiate Between Benign and Malignant Prostate Lesions. Eur J Radiol (2019) 114:38–44. 10.1016/j.ejrad.2019.02.032 31005174

[27] Park YW Oh J You SC Han K Ahn SS Choi YS. Radiomics and Machine Learning may Accurately Predict the Grade and Histological Subtype in Meningiomas Using Conventional and Diffusion Tensor Imaging. Eur Radiol (2019) 29:4068–76. 10.1007/s00330-018-5830-3 30443758

[28] Su C Jiang J Zhang S Shi J Xu K Shen N. Radiomics Based on Multicontrast MRI can Precisely Differentiate Among Glioma Subtypes and Predict Tumour-Proliferative Behaviour. Eur Radiol (2019) 29:1986–96. 10.1007/s00330-018-5704-8 30315419

[29] Yang C Huang M Li S Chen J Yang Y Qin N. Radiomics Model of Magnetic Resonance Imaging for Predicting Pathological Grading and Lymph Node Metastases of Extrahepatic Cholangiocarcinoma. Cancer Lett (2020) 470:1–7. 10.1016/j.canlet.2019.11.036 31809800

[30] Kim M Jung SY Park JE Jo Y Park SY Nam SJ. Diffusion- and Perfusion-Weighted MRI Radiomics Model may Predict Isocitrate Dehydrogenase (IDH) Mutation and Tumor Aggressiveness in Diffuse Lower Grade Glioma. Eur Radiol (2020) 30:2142–51. 10.1007/s00330-019-06548-3 31828414

[31] Wang C Li H Jiaerken Y Huang P Sun L Dong F. Building CT Radiomics-Based Models for Preoperatively Predicting Malignant Potential and Mitotic Count of Gastrointestinal Stromal Tumors. Transl Oncol (2019) 12:1229–36. 10.1016/j.tranon.2019.06.005 PMC6614115 31280094

[32] Zhang Z Jiang H Chen J Wei Y Cao L Ye Z. Hepatocellular Carcinoma: Radiomics Nomogram on Gadoxetic Acid-Enhanced MR Imaging for Early Postoperative Recurrence Prediction. Cancer Imaging (2019) 19:22. 10.1186/s40644-019-0209-5 31088553 PMC6518803

[33] Ford J Dogan N Young L Yang F. Quantitative Radiomics: Impact of Pulse Sequence Parameter Selection on MRI-Based Textural Features of the Brain. Contrast Media Mol Imaging (2018) 2018:1729071. 10.1155/2018/1729071 30154684 PMC6091359

[34] Lecler A Duron L Balvay D Savatovsky J Berges O Zmuda M. Combining Multiple Magnetic Resonance Imaging Sequences Provides Independent Reproducible Radiomics Features. Sci Rep (2019) 9:2068. 10.1038/s41598-018-37984-8 30765732 PMC6376058

[35] Liu X Song M Tao D Liu Z Zhang L Chen C. Random Forest Construction With Robust Semisupervised Node Splitting. IEEE Trans Image Process (2015) 24:471–83. 10.1109/TIP.2014.2378017 25494503

[36] Gebreyohannes YK Wozniak A Zhai ME Wellens J Cornillie J Vanleeuw U. Robust Activity of Avapritinib, Potent and Highly Selective Inhibitor of Mutated KIT, in Patient-derived Xenograft Models of Gastrointestinal Stromal Tumors. Clin Cancer Res (2019) 25:609–18. 10.1158/1078-0432.CCR-18-1858 30274985

[37] Joensuu H Wardelmann E Sihto H Eriksson M Sundby Hall K Reichardt A. Effect of KIT and PDGFRA Mutations on Survival in Patients With Gastrointestinal Stromal Tumors Treated With Adjuvant Imatinib: An Exploratory Analysis of a Randomized Clinical Trial. JAMA Oncol (2017) 3:602–9. 10.1001/jamaoncol.2016.5751 PMC5470395 28334365

