# Supplementary material for: MRI-Based Radiomics Models for Predicting Risk Classification of Gastrointestinal Stromal Tumors
Source: Front Oncol. 2021 May 10;11:631927. doi: 10.3389/fonc.2021.631927 (PMC8141866; doi:10.3389/fonc.2021.631927)
Supplement: Supplementary file 1 [file Table_1.docx]

**Supplementary Table 1.** Optimal 30 features subset for identifying the risk of GISTs in T1WI

| **Radiomic Features** | **Low-risk** | **Intermediate-risk** | **High-risk** | **Feature_imoportances** |
| --- | --- | --- | --- | --- |
| GreyLevelNonuniformity_angle135_offset4 | 14.41(7.27, 26.95) | 62.64(15.58, 141.75) | 192.50(94.43, 718.93) | 20 |
| GreyLevelNonuniformity_angle45_offset7 | 14.79(7.24, 26.90) | 63.21(15.74, 142.90) | 193.39(94.51, 724.12) | 4.403522 |
| GreyLevelNonuniformity_angle90_offset7 | 14.49(7.27, 26.75) | 62.68(15.67, 142.16) | 192.74(94.58, 721.14) | 4.284136 |
| GreyLevelNonuniformity_angle135_offset1 | 13.53(6.97, 25.63) | 60.77(15.21, 137.30) | 191.02(89.71, 657.40) | 4.271273 |
| GreyLevelNonuniformity_AllDirection_offset7 | 14.58(7.25, 26.81) | 62.86(15.66, 142.58) | 192.81(94.67, 723.82) | 3.862838 |
| RunLengthNonuniformity_angle90_offset4 | 687.21(411.90, 1566.04) | 3772.24(1259.17, 12561.15) | 11654.00(7562.64, 61696.96) | 3.85888 |
| GreyLevelNonuniformity_angle0_offset7 | 14.32(7.23, 26.60) | 62.71(15.55, 142.16) | 192.28(94.36, 718.80) | 3.801806 |
| RunLengthNonuniformity_AllDirection_offset1 | 669.17(375.45, 1441.14) | 3254.32(1131.42, 11551.97) | 11637.40(6705.59, 53977.12) | 3.714233 |
| RunLengthNonuniformity_AllDirection_offset7 | 707.26(424.90, 1615.56) | 3948.83(1269.51, 12984.56) | 11772.00(7828.10, 65779.78) | 3.670513 |
| GreyLevelNonuniformity_angle135_offset7 | 14.73(7.27, 26.99) | 62.84(15.67, 143.09) | 192.84(95.22, 731.20) | 3.442013 |
| RunLengthNonuniformity_angle135_offset4 | 708.01(420.46, 1616.32) | 3846.35(1270.38, 12854.84) | 11744.50(7738.94, 65592.84) | 3.073404 |
| RunLengthNonuniformity_angle135_offset1 | 684.28(386.72, 1498.02) | 3364.96(1168.32, 11817.06) | 11456.50(6848.71, 56934.10) | 2.930832 |
| RunLengthNonuniformity_angle90_offset7 | 708.01(421.61, 1610.98) | 3914.68(1264.46, 12894.93) | 11774.90(7788.82, 64096.20) | 2.908609 |
| VolumeCount | 713.00(435.80, 1657.70) | 4212.00(1292.00, 13659.20) | 12175.00(8170.20, 70049.40) | 2.734576 |
| RunLengthNonuniformity_angle45_offset7 | 702.04(432.91, 1628.82) | 4003.27(1278.73, 13019.21) | 11867.70(7864.40, 64744.54) | 2.602361 |
| GreyLevelNonuniformity_angle0_offset4 | 14.53(7.11, 26.37) | 62.21(15.53, 140.90) | 191.44(93.05, 703.07) | 2.449746 |
| GreyLevelNonuniformity_angle90_offset4 | 14.27(7.17, 26.51) | 61.95(15.55, 140.65) | 192.15(93.39, 703.25) | 2.431745 |
| GreyLevelNonuniformity_angle45_offset4 | 14.38(7.08, 26.45) | 62.46(15.41, 141.82) | 192.53(93.59, 707.23) | 2.408516 |
| GreyLevelNonuniformity_angle90_offset1 | 13.75(6.95, 25.40) | 59.77(14.84, 134.47) | 190.41(88.29, 622.79) | 1.786043 |
| RunLengthNonuniformity_AllDirection_offset4 | 699.08(414.97, 1579.62) | 3794.58(1251.27, 12643.72) | 11678.60(7609.05, 63354.48) | 1.167194 |
| Maximum3DDiameter | 22.00(15.81, 28.38) | 38.12(26.54, 53.37) | 66.29(50.32, 80.38) | 1.132107 |
| RunLengthNonuniformity_angle135_offset7 | 711.00(429.62, 1625.56) | 4046.48(1271.88, 13170.93) | 11766.10(7884.50, 67910.04) | 0.777655 |
| RunLengthNonuniformity_angle0_offset4 | 699.06(417.77, 1550.59) | 3731.72(1215.24, 12465.96) | 11589.30(7473.52, 63588.38) | 0.757561 |
| GreyLevelNonuniformity_AllDirection_offset4 | 14.44(7.15, 26.57) | 62.31(15.52, 141.28) | 192.15(93.61, 708.12) | 0.755643 |
| RunLengthNonuniformity_AllDirection_offset7_SD | 115.03(20.22, 383.14) | 1815.38(193.97, 32167.12) | 43969.00(4135.15, 1233034.40) | 0.652629 |
| GreyLevelNonuniformity_AllDirection_offset1 | 13.77(6.92, 25.61) | 60.26(14.99, 135.69) | 190.05(88.92, 639.28) | 0.435137 |
| FrequencySize | 712.00(434.80, 1656.70) | 4211.00(1290.30, 13657.90) | 12174.00(8168.40, 70048.40) | 0.370727 |
| GreyLevelNonuniformity_angle45_offset1 | 13.84(6.94, 25.82) | 60.79(15.11, 137.24) | 191.11(89.85, 647.63) | 0.254029 |
| GreyLevelNonuniformity_angle0_offset1 | 13.76(6.87, 25.59) | 59.69(14.79, 133.74) | 187.67(87.84, 629.31) | 0.252613 |
| RunLengthNonuniformity_angle45_offset4 | 702.04(413.10, 1585.52) | 3828.04(1260.29, 12692.89) | 11726.80(7661.09, 62539.74) | 0.067053 |

**Supplementary Table 2.** Optimal 30 features subset for identifying the risk of GISTs in T2WI

| **Radiomic Features** | **Low-risk** | **Intermediate-risk** | **High-risk** | **Feature_imoportances** |
| --- | --- | --- | --- | --- |
| RunLengthNonuniformity_angle0_offset7 | 1070.08(501.86, 1824.99) | 4397.52(1998.88, 8505.71) | 15610.40(8691.84, 51848.72) | 4.429353 |
| RunLengthNonuniformity_angle45_offset4 | 1045.37(502.46, 1807.16) | 4275.34(1983.24, 8495.35) | 15484.70(8709.20, 51398.46) | 4.369383 |
| RunLengthNonuniformity_angle0_offset1 | 1051.01(500.82, 1803.14) | 4357.04(1957.14, 8341.00) | 15456.50(8476.63, 50861.36) | 4.357034 |
| RunLengthNonuniformity_AllDirection_offset7 | 1067.86(508.45, 1845.43) | 4421.70(1999.13, 8682.76) | 15732.30(8767.52, 52361.40) | 4.341686 |
| RunLengthNonuniformity_angle135_offset4 | 1061.17(501.57, 1841.24) | 4382.79(1980.16, 8587.07) | 15505.60(8715.66, 51756.46) | 4.326928 |
| GreyLevelNonuniformity_AllDirection_offset1_SD | 0.01(0.00, 0.05) | 0.18(0.04, 0.73) | 5.51(0.20, 59.70) | 4.315252 |
| GreyLevelNonuniformity_AllDirection_offset1 | 9.96(6.28, 24.50) | 58.82(26.23, 106.02) | 195.27(81.80, 660.25) | 4.20543 |
| RunLengthNonuniformity_angle90_offset1 | 1052.01(497.02, 1821.82) | 4368.03(1954.09, 8334.22) | 15401.70(8587.72, 50761.66) | 4.187022 |
| RunLengthNonuniformity_angle45_offset1 | 989.13(482.03, 1721.65) | 4067.92(1902.22, 7806.43) | 14351.20(8184.12, 46853.08) | 4.103704 |
| GreyLevelNonuniformity_AllDirection_offset4 | 10.26(6.47, 25.03) | 59.99(26.84, 110.47) | 201.82(83.80, 690.22) | 4.102722 |
| GreyLevelNonuniformity_angle0_offset7 | 10.31(6.44, 25.16) | 60.28(26.94, 110.79) | 203.81(83.74, 695.04) | 4.001219 |
| RunLengthNonuniformity_angle135_offset1 | 1001.64(480.58, 1750.82) | 4118.95(1892.30, 7756.55) | 14569.00(8180.80, 47269.24) | 3.641758 |
| GreyLevelNonuniformity_angle90_offset1 | 9.95(6.03, 24.46) | 58.73(26.10, 105.40) | 193.63(81.69, 651.29) | 3.521618 |
| RunLengthNonuniformity_AllDirection_offset4 | 1045.90(503.71, 1815.20) | 4316.79(1976.35, 8432.36) | 15389.60(8653.12, 51098.68) | 3.126417 |
| RunLengthNonuniformity_angle90_offset7 | 1058.21(508.67, 1850.48) | 4424.08(1988.29, 8705.03) | 15619.50(8767.20, 52103.60) | 2.744339 |
| RunLengthNonuniformity_angle90_offset4 | 1036.54(500.97, 1814.08) | 4316.19(1974.84, 8380.00) | 15225.30(8691.65, 50917.98) | 2.526713 |
| RunLengthNonuniformity_angle0_offset4 | 1040.51(509.86, 1804.85) | 4292.82(1967.15, 8266.99) | 15342.90(8495.98, 50321.68) | 2.461854 |
| GreyLevelNonuniformity_angle90_offset4 | 10.25(6.48, 25.00) | 60.05(26.87, 109.97) | 200.98(83.72, 688.80) | 2.429414 |
| GreyLevelNonuniformity_angle45_offset7 | 10.35(6.44, 25.24) | 60.31(27.11, 111.94) | 204.50(84.53, 698.17) | 2.419188 |
| GreyLevelNonuniformity_angle0_offset4 | 10.26(6.40, 24.97) | 59.89(26.88, 109.77) | 201.84(83.32, 686.14) | 2.015765 |
| GreyLevelNonuniformity_AllDirection_offset4_SD | 0.00(0.00, 0.02) | 0.03(0.01, 0.11) | 0.67(0.07, 9.20) | 2.003537 |
| GreyLevelNonuniformity_angle0_offset1 | 9.93(6.32, 24.38) | 58.56(25.80, 104.61) | 193.95(81.34, 655.12) | 1.703474 |
| GreyLevelNonuniformity_AllDirection_offset7_SD | 0.00(0.00, 0.01) | 0.04(0.01, 0.12) | 0.65(0.06, 5.44) | 1.636022 |
| GreyLevelNonuniformity_angle45_offset1 | 10.01(6.37, 24.56) | 58.99(26.66, 107.14) | 196.27(81.93, 665.99) | 1.087118 |
| RunLengthNonuniformity_angle135_offset7 | 1073.06(511.64, 1862.04) | 4485.35(1991.50, 8832.36) | 15866.30(8804.81, 52862.34) | 0.708994 |
| RunLengthNonuniformity_angle45_offset7 | 1070.08(511.64, 1844.23) | 4379.85(2017.84, 8687.97) | 15833.00(8806.21, 52630.96) | 0.645006 |
| RunLengthNonuniformity_AllDirection_offset1 | 9.96(6.28, 24.50) | 58.82(26.23, 106.02) | 195.27(81.80, 660.25) | 0.51639 |
| FrequencySize | 1089.00(520.10, 1893.30) | 4571.00(2064.50, 9108.70) | 16487.00(8985.20, 55596.60) | 0.513143 |
| RunLengthNonuniformity_AllDirection_offset7_SD | 55.02(18.70, 275.56) | 1159.60(231.74, 7632.77) | 23425.80(5589.69, 193139.40) | 0.16916 |
| VolumeCount | 1090.00(521.10, 1895.00) | 4572.00(2065.50, 9109.70) | 16488.00(8986.20, 55597.60) | 0.123502 |

**Supplementary Table 3.** Optimal 30 features subset for identifying the risk of GISTs in ADC

| **Radiomic Features** | **Low-risk** | **Intermediate-risk** | **High-risk** | **Feature_imoportances** |
| --- | --- | --- | --- | --- |
| MinIntensity | 930.00(498.30, 1154.80) | 286.00(0.00, 513.60) | 0.00(0.00, 251.00) | 4.91704662 |
| GreyLevelNonuniformity_AllDirection_offset1 | 6.89(2.05, 11.32) | 23.97(10.05, 82.26) | 94.18(13.24, 224.18) | 4.89629981 |
| RunLengthNonuniformity_angle0_offset7 | 502.01(130.12, 1061.34) | 1961.17(800.26, 7242.24) | 7825.37(1487.01, 26515.74) | 4.68521203 |
| VolumeCC | 8.60(4.47, 14.04) | 36.53(18.33, 85.74) | 139.27(42.82, 541.18) | 4.63074134 |
| RunLengthNonuniformity_angle0_offset4 | 487.17(130.12, 1037.91) | 1944.38(804.64, 7088.84) | 7737.40(1476.94, 26420.38) | 4.62198279 |
| RunLengthNonuniformity_angle0_offset1 | 493.08(122.48, 962.43) | 1823.47(786.32, 6160.42) | 7330.68(1395.22, 25499.16) | 4.55504182 |
| GreyLevelNonuniformity_angle135_offset1 | 6.98(2.03, 10.51) | 23.98(9.81, 83.26) | 94.83(13.32, 233.21) | 4.52974869 |
| SurfaceVolumeRatio | 304.07(267.41, 416.08) | 187.65(140.67, 277.83) | 123.57(73.92, 190.58) | 4.47441062 |
| VolumeMM | 8601.90(4467.38, 14042.13) | 36533.70(18332.19, 85743.74) | 139272.00(42820.36, 541178.20) | 4.41102829 |
| RunLengthNonuniformity_AllDirection_offset7 | 502.01(130.12, 1058.07) | 1957.48(808.19, 7244.77) | 7871.99(1499.96, 26415.24) | 4.11007683 |
| RunLengthNonuniformity_angle90_offset7 | 499.03(130.12, 1056.59) | 1953.27(770.94, 7206.08) | 7830.21(1501.11, 26414.86) | 4.05834548 |
| RunLengthNonuniformity_angle45_offset4 | 496.05(130.12, 1041.75) | 1956.24(854.95, 7120.62) | 7829.18(1499.50, 26512.70) | 3.9074353 |
| GreyLevelNonuniformity_angle90_offset1 | 6.87(2.04, 10.26) | 23.74(10.37, 80.80) | 93.77(13.10, 215.50) | 3.23617136 |
| GreyLevelNonuniformity_angle0_offset1 | 6.89(2.01, 11.16) | 23.99(10.82, 81.50) | 93.53(13.05, 220.76) | 2.93848127 |
| RunLengthNonuniformity_angle90_offset4 | 493.08(130.12, 1042.63) | 1949.56(785.01, 7046.25) | 7766.62(1484.43, 26395.14) | 2.62096046 |
| GreyLevelNonuniformity_angle45_offset1 | 6.83(2.07, 13.86) | 24.18(9.84, 83.48) | 94.60(13.38, 227.24) | 2.60096162 |
| RunLengthNonuniformity_angle135_offset4 | 502.01(130.12, 1040.56) | 1939.81(842.67, 7180.28) | 7857.68(1490.38, 26413.28) | 2.51171046 |
| FrequencySize | 506.00(131.10, 1074.90) | 2024.00(970.80, 7585.70) | 8227.00(1521.00, 28767.80) | 2.13884694 |
| GreyLevelNonuniformity_angle0_offset4 | 6.84(2.10, 14.26) | 25.00(17.21, 87.48) | 97.46(13.52, 279.91) | 2.03584533 |
| RunLengthNonuniformity_angle90_offset1 | 475.45(121.93, 932.79) | 1799.03(757.99, 6057.13) | 7117.54(1387.23, 25291.50) | 1.95560086 |
| GreyLevelNonuniformity_angle90_offset4 | 6.87(2.10, 14.31) | 24.88(18.36, 87.28) | 98.91(13.53, 281.15) | 1.89836353 |
| RunLengthNonuniformity_angle45_offset1 | 484.23(127.15, 985.36) | 1859.67(817.08, 6403.44) | 7408.38(1430.18, 25983.12) | 1.52884186 |
| VolumeCount | 507.00(132.10, 1075.90) | 2025.00(972.50, 7586.70) | 8228.00(1525.20, 31267.60) | 1.52797417 |
| RunLengthNonuniformity_angle135_offset7 | 505.00(130.12, 1057.17) | 1960.46(856.94, 7267.06) | 7904.90(1507.26, 26284.98) | 1.17615437 |
| RunLengthNonuniformity_angle135_offset1 | 489.11(118.17, 974.05) | 1851.42(798.38, 6372.72) | 7384.24(1434.13, 25978.48) | 0.9076633 |
| SurfaceArea | 2570.90(1883.14, 3766.31) | 6855.50(4793.02, 12087.20) | 17209.20(7519.24, 40112.14) | 0.83090883 |
| RunLengthNonuniformity_AllDirection_offset1 | 485.47(122.43, 963.66) | 1830.96(789.94, 6248.43) | 7310.21(1411.69, 25688.04) | 0.80431287 |
| RunLengthNonuniformity_AllDirection_offset4 | 494.58(130.12, 1040.71) | 1942.00(821.81, 7109.00) | 7797.72(1487.81, 26435.40) | 0.6682249 |
| LongRunEmphasis_angle135_offset4 | 1.39(1.03, 1.58) | 2.71(1.77, 25.13) | 14.13(2.08, 21.07) | 0.49876571 |
| RunLengthNonuniformity_angle45_offset7 | 502.01(130.12, 1057.17) | 1975.27(804.63, 7263.72) | 7927.46(1504.48, 26445.24) | 0.12098609 |
